# Impaired Granuloma Formation in Sepsis: Impact of Monocytopenia

**DOI:** 10.1371/journal.pone.0158528

**Published:** 2016-07-21

**Authors:** Julie Alingrin, Benjamin Coiffard, Julien Textoris, Pauline Belenotti, Aurélie Daumas, Marc Leone, Jean-Louis Mege

**Affiliations:** 1 Unité de Recherche sur les Maladies Infectieuses Tropicales et Emergentes, UMR 63, CNRS 7278, IRD 198, INSERM U1095, Aix-Marseille Université, Marseille, France; 2 Service d’Anesthésie et de Réanimation, Hôpital Nord, Assistance Publique-Hôpitaux de Marseille, Aix-Marseille Université, Marseille, France; 3 Service de Médecine Interne, Gériatrie et Thérapeutique, Hôpital de la Timone, Assistance Publique-Hôpitaux de Marseille, Aix-Marseille Université, Marseille, France; INSERM, FRANCE

## Abstract

Granulomas are a collection of immune cells considered to be protective in infectious diseases. The *in vitro* generation of granulomas is an interesting substitution to invasive approaches of granuloma study. The monitoring of immune response through the determination of *in vitro* granuloma formation in patients with severe sepsis may be critical to individualize treatments. We compared the *in vitro* generation of granulomas by co-culturing circulating mononuclear cells from 19 patients with severe sepsis, 9 patients cured from Q fever and 12 healthy subjects as controls, and Sepharose beads coated either with BCG or *Coxiella burnetii* extracts to analyze both immune and innate granulomas, respectively. We showed that the great majority of patients with severe sepsis were unable to form granulomas in response to BCG and *C*. *burnetii* extracts whereas more than 80% of healthy controls and patients cured from Q fever formed granulomas. We also found that monocytopenia and defective production of tumor necrosis factor were associated with reduced formation of granulomas in patients with severe sepsis even if TNF did not seem to be involved in the defective granuloma formation. Taken together, these results suggest that the deficiency of granuloma formation may be a measurement of altered recruitment and activation of monocytes and lymphocytes in patients with severe sepsis.

## Introduction

Granulomas are a tissue collection of macrophages and lymphocytes, which are generated in response to various microorganisms [1], toxic molecules and foreign materials [2]. The first step of granuloma formation is the recruitment of macrophages and blood-derived myeloid cells, which is completed by the recruitment of activated T cells. Macrophages within granulomas undergo a maturation program leading to the formation of multinucleated giant cells (MGCs) and foamy cells [3,4]. The formation of granulomas is conditioned by the cytokine context. Indeed, type 1 cytokines such as interferon (IFN)-γ, interleukin (IL)-12, and tumor necrosis factor (TNF) are necessary for the formation of granulomas in response to bacteria [5]. The granulomas evolve from “innate granulomas” that do not require T cells to “immune granulomas” in which T cells are required [6].

The study of tissue granulomas requires invasive methods that are not convenient for the investigation of patients. A method was recently developed to generate granulomas *in vitro* using the co-culture of peripheral blood mononuclear cells (PBMCs) and Sepharose beads coated with bacterial extracts from BCG [7] or *Coxiella burnetii* (CB) [8]. The monocytes migrate to the beads and maturate into macrophages and MGCs in the presence of lymphocytes [8]. Hence, the *in vitro* assay of granuloma formation measures the activation and the ability of monocytes to migrate towards the source of infection and could be used in clinics. For instance, this assay has been used to study the ability of invasive *Escherichia coli* from patients with Crohn disease to elicit the aggregation of macrophages and lymphocytes [9]. A similar assay combined with high-content screening technology allows investigating anti-tuberculous compound activities [10]. In brain-injured patients who develop nosocomial pneumonia, PBMCs generate fewer mature granulomas than those of controls in response to BCG [11]. PBMCs from a large proportion of patients with Q fever endocarditis are unable to form granulomas in the presence of beads coated with CB extracts. This is related to altered recruitment of monocytes since the distance covered by monocytes was lower in Q fever endocarditis than in controls [12]. Hence, the *in vitro* assay can mimic the lack of granulomas found in patients with chronic evolution of Q fever and suggests that impaired monocyte migration is involved in defective formation of granulomas. Taken together, these data suggest that the *in vitro* formation of granulomas could be a new method to assess recruitment and activation of immune cells in patients.

Severe sepsis is defined as the combination of a systemic inflammatory response syndrome and suspected infection with at least one organ failure including hypotension, respiratory failure, coma, liver failure, thrombocytopenia and acute renal failure [13]. It is characterized by an uncontrolled inflammatory response that leads to immune system failure including a loss of delayed hypersensitivity, an inability to clear infections and a predisposition to nosocomial infections [14]. Some cytokines such as circulating TNF have been correlated with the prognosis of severe sepsis [15] and such findings have served as a basis for immunotherapy of sepsis targeting TNF. Unfortunately, this strategy was unsuccessful [16]. Few molecules have been proposed to assess the prognosis of severe sepsis and to identify patients who can benefit from innovative treatments. For instance, the expression of HLA-DR is used before the administration of granulocyte-macrophage colony-stimulating factor in patients with a defective immune response [17]. Although these tools investigate some features of the immune response, we need bioassays that enable us to assess the activation of immune cells in patients to adapt immune therapy.

Here, we assessed the ability of PBMCs from patients with severe sepsis to generate granulomas. To exonerate our data from the constraints of prior immunization, we compared granuloma formation in response to BCG and CB (a majority *vs*. a minority of patients specifically immunized). We found that 13 of 19 patients with severe sepsis were unable to form granulomas, suggesting severe impairment of their ability to recruit and activate immune cells. We also demonstrated a relationship between the impairment of granuloma formation and monocytopenia.

## Materials and Methods

### Study population

Patient recruitment was provided from an ancillary study to the “De-escalation of Empirical Antibiotics in Severe Sepsis” project (Comité de Protection des Personnes Sud Méditerranée no. 2011-002297-22) [18]. Patients were enrolled from February 1^st^, 2012, to April 8^th^, 2013, at the polyvalent intensive care unit (ICU) (15 beds) of the North Hospital (968 beds), Marseille, France. Written informed consent was obtained from the patients and their relatives. Eligibility criteria were the presence of severe sepsis requiring an empirical antimicrobial treatment. At the time of blood collection, we collected demographic variables, causes of ICU admission, source of infection, use of vasopressors, need for mechanical ventilation, lymphocyte and monocyte blood counts determined by numeration formula of leukocytes the first day of sepsis (cells/l), the simplified acute physiology score (SAPS) 2 [19] and the sequential organ failure assessment (SOFA) score reflecting the number of organ failures [20]. On ICU discharge, we collected the number of ICU days, the number of days with mechanical ventilation, the number of days with vasopressors and the ICU mortality rate. A total of 19 patients with severe sepsis (17 males and two females) were enrolled in the study with a median age of 57 years [49–66] with the lungs as primary infection site (16 patients) (Table 1). Lymphopenia and monocytopenia are defined as cell counts lower than 1 × 10⁹ cells/l and 0.2 × 10⁹ cells/l, respectively. An historical cohort of 21 individuals as controls including 12 healthy donors and 9 patients with cured Q fever was defined on clinical (history of acute Q fever) and serological recovery (residual levels of anti-*C*. *burnetii* antibodies (Abs)).

**Table 1:** Patient features.

Variables	Values
**Clinical features**	
Patients (males/females)	19 (17/2)
Age [years]	57 [49–66]
SOFA	8 [6–10]
SAPS II	38 [30–48]
Number of ICU days	12 [8–17]
Mechanical ventilation (days)	8 [5–13]
**Causes of ICU admission (%)**	
Sepsis	7 (37)
Head trauma	4 (21)
Coma	2 (11)
Stroke	2 (11)
Active bleeding	2 (11)
Acute respiratory failure	2 (11)
**Sources of infection (%)**	
Lung	16 (84)
Urinary tract	2 (11)
Abdomen	1 (5.2)
**Microbiological findings (%)**	
MSSA	7 (37)
*Streptococcus pneumoniae*	3 (16)
Other Gram positive bacteria	1 (5)
*Enterobacteria*	5 (26)
Other Gram negative bacteria	5 (26)

SOFA: sequential organ failure assessment; SAPS: simplified acute physiology score; ICU: intensive care unit; MSSA: Methicillin susceptible *Staphylococcus aureus*. Results are expressed as absolute number and percentage or median and interquartiles, as required.

### PBMC isolation

The EDTA-anticoagulated blood samples (5 ml) were withdrawn the day of empirical antibiotic onset. The PBMCs were isolated after centrifugation on Ficoll cushion, as previously described [21]. The PBMCs were then suspended (about 5 × 10⁶ cells/ml) in RPMI 1640 containing 20% fetal calf serum (FCS, Invitrogen) and 10% dimethyl sulfoxide and conserved at -80°C.

### Bacteria and bead preparation

BCG (*Mycobacterium bovis*, American Type Culture Collection, stub 35734) was cultured on agarose (Middlebrook 7H10 agar medium). *C*. *burnetii* (Nine Mile strain RSA496) was cultured as previously described [22]. The bacteria (10^9^ per assay) were sonicated in a coupling buffer (NaHCO_3_ 0.1 M pH 8.3 with NaCl 0.5 M) and their protein content was determined by Bradford’s method as previously described [12]. Activated 4B Sepharose beads (about 4 × 10^4^ beads, 40–100 μm diameter, GE Healthcare) were coated with bacterial extracts (0.5 mg of proteins). The coupling efficiency was determined by measuring the protein content of supernatants.

### Granuloma formation

The formation of granulomas was studied using a modified method adapted from Delaby *et al*. to investigate patients with severe sepsis [12]. For that purpose, different concentrations of PBMCs (1 × 10^5^ and 2.5 × 10^5^ per well) were incubated in 96-well microplates in the presence of 50 or 100 beads coated with BCG or CB in 200 μl of RPMI 1640 containing 10% FCS and antibiotics at 37°C. The formation of granulomas was measured after 3, 6 and 9 days in duplicates and evaluated by reverse microscopy. Only beads that were completely covered by cells were considered granulomas and the results were expressed as the percentage of granulomas. The reproducibility was assessed in 3 individuals who were assayed 3 times with similar results. To assess the impact of freezing on the ability to forming granulomas, PBMCs from three individuals were frozen or immediately used, and the formation of granulomas was measured. In some experiments, PBMCs from patients who did not form granulomas were incubated with beads coated with BCG or CB extracts and 10 ng/ml of human recombinant TNF (R&D Systems) while control PBMCs were incubated with coated beads and 10 ng/ml monoclonal Ab (mAb) directed against TNF (Adalimumab kindly provided by Professor J. Roudier, Marseille) and granuloma formation was measured. Crude data are available in the S1 Table.

### Cytokine measurement

PBMCs (2.5 × 10^5^ cells/well) were incubated with beads coated with bacterial extracts (50 beads/well) for 1 day of culture. Each experiment was performed in duplicate. Cell supernatants were collected and TNF and IL-10 were assayed in duplicates (100 μl each) using specific immunoassays (R&D Systems). The results were expressed as pg/ml. The detection limits of immunoassays were of 5.5 and 3.9 pg/ml for TNF and IL-10, respectively. The intra- and interspecific coefficients of variation ranged from 5% to 10%. Crude data are available in S2 Table.

### Statistical analysis

Quantitative data are presented as the medians and interquartile ranges [IQRs], and mean and standard deviation. Qualitative data are presented as absolute counts and percentages. Differences between groups were tested with the Student *t* test and a cutoff value of 0.05 was chosen to consider a difference to be statistically significant. Data analysis and plots were performed using R software (v.3.1.2) with “stats” and “*ggplot2”* libraries (Bioconductor software suite).

## Results

### *In vitro* generation of granulomas in patients with severe sepsis

In a first series of experiments, we defined the experimental conditions of granuloma formation using three healthy controls. We found that the culture of 2.5 × 10^5^ PBMCs with 50 BCG- or CB-coated beads in 96-well microplates led to the formation of 75% and 90% of granulomas after 6 and 9 days, respectively. We also measured the effect of PBMC freezing on granuloma formation. The ability to form granuloma was not significantly different in unfrozen and frozen PBMCs (92 ± 5% *vs*. 80 ± 7% after 9 days, respectively). In addition, we assayed the ability of three control PBMCs to form granulomas three times. The variations in granuloma formation did not exceed 10%. Hence, these conditions, which reduced blood sampling and provided a reproducible assay for routine investigation of frozen PBMCs from septic patients, were used in the subsequent experiments.

**Fig 1:**
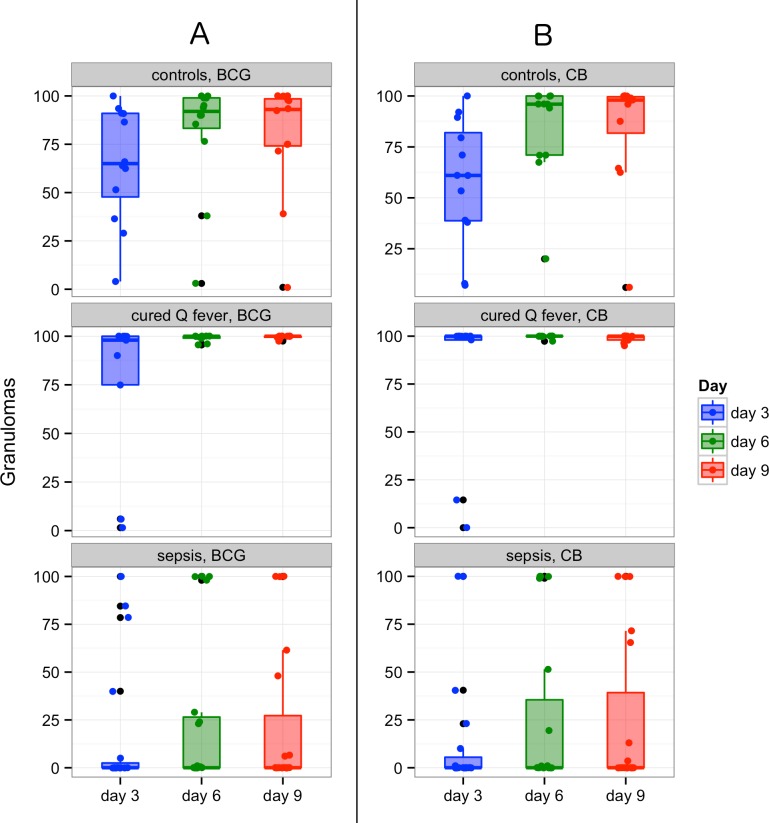
Granuloma formation in controls, cured Q fever and patients with severe sepsis. PBMCs (2.5 × 10^5^) were cultured during 3, 6 and 9 days in the presence of 50 beads coated with BCG (A) or CB (B) extracts. PBMCs were isolated from controls (upper part), patients cured from Q fever (medium part) and patients with severe sepsis (lower part). Granuloma formation is expressed as the percentage of beads entirely covered by PBMCs. The boxplots represent the medians with the first and third quartiles. The whiskers represent the highest value that is within 1.5* IQR*. Data beyond the end of the whiskers are outliers and plotted as black points. Color points represent the mean value of the duplicates.

Then, we investigated the formation of granulomas in patients with severe sepsis. Severe sepsis was characterized by a dramatic decrease in granuloma formation as compared with healthy controls and individuals with cured Q fever. Indeed, after 3 days of culture of PBMCs with BCG-coated beads, the formation of granulomas was low (16 ± 33%) in septic patients whereas it already reached 65 ± 30% in healthy controls (p < 0.001). Note that one healthy control was unable to form granulomas and two subjects form only a small number of granulomas. The defective formation of granulomas in septic patients was not due to a delayed formation of granulomas since it remained lower in the septic patients than in the healthy controls after 6 (25 ± 41% *vs*. 81 ± 30%, p < 0.001) and 9 days (22 ± 39% *vs*. 79 ± 30%, p < 0.001). It was clearly related to severe sepsis because the patients cured from Q fever were fully able to form BCG-specific granulomas; the percentage was close to 100% (75 ± 41%; 98 ±2%; 99 ±1%; p < 0.001 at days 3, 6 and 9, respectively) (Fig 1A). The deficient formation of granulomas did not affect all the patients with severe sepsis because six among the 19 (31%) patients kept the ability to form granulomas. Note that granuloma formation was delayed in 3 of these patients. The patients with SOFA ≥ 7 tended to have a defective granuloma formation, but no significant correlation was found (S1 Fig). No significant difference was found between the patients with and without granuloma formation (S3 Table).

Because the deficiency of granuloma formation in severe sepsis was related to the immune granulomas, we wondered if innate granulomas were also affected. We determined the ability of PBMCs from patients with severe sepsis to form innate granulomas using beads covered with CB extracts. We showed that the patients with severe sepsis were unable to form innate granulomas (14 ± 32%; 25 ± 42%; 24 ± 40%) compared with the healthy controls (58 ± 31%; 84 ± 24%; 84 ± 28%; p < 0.001) and with the patients cured from Q fever (79 ± 41%; 99 ± 1%; 98 ± 2%; p < 0.001) at days 3, 6, and 9, respectively. Note that the patient who did not form granulomas in response to BCG extracts was unable to mount a granuloma response to CB extracts. The level of the deficiency in patients with severe sepsis was similar to that found for immune granulomas (Fig 1B). Taken together, these results showed that the patients with severe sepsis exhibited a defective formation of innate and immune granulomas.

### Mechanisms of defective granuloma formation in septic patients

**Fig 2:**
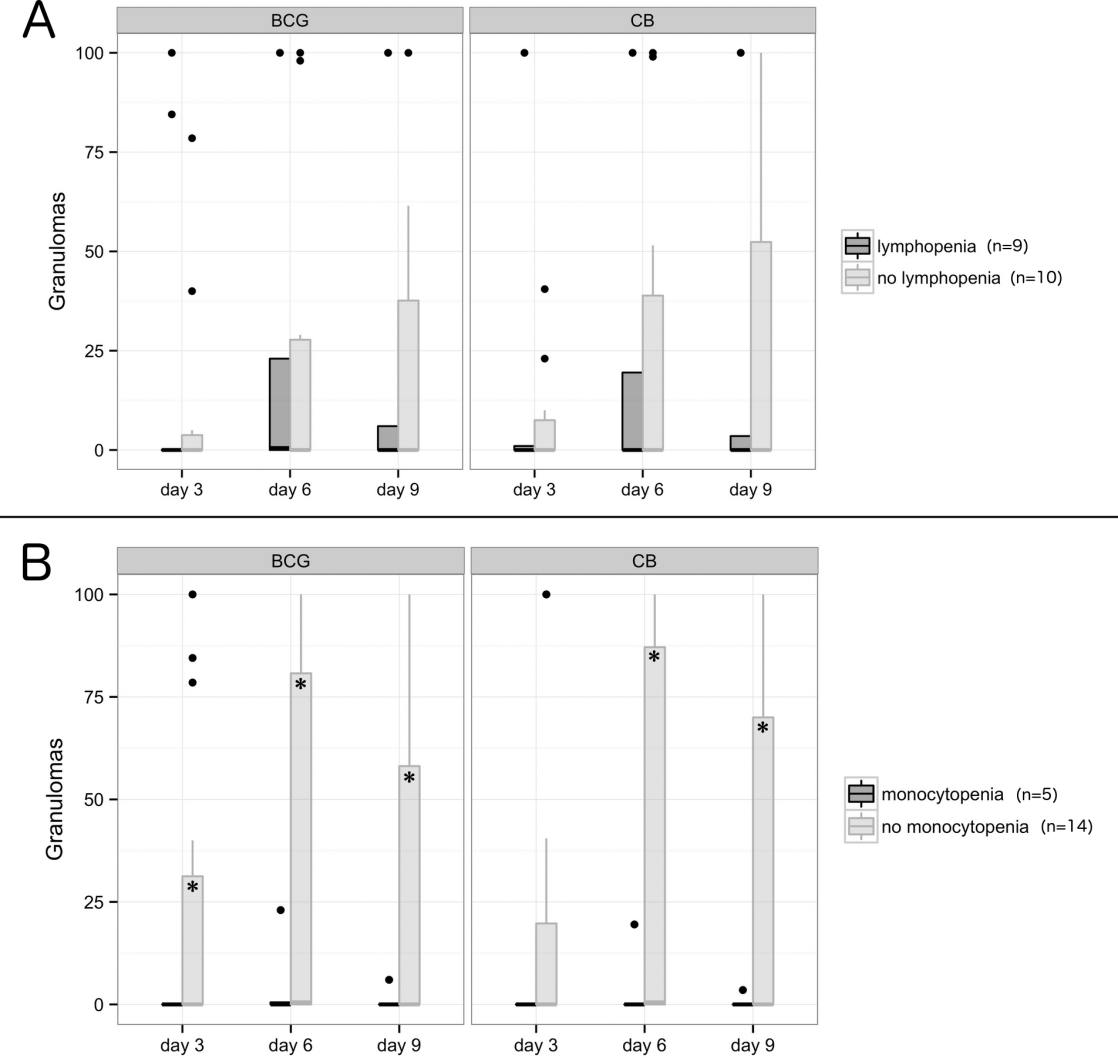
Granuloma formation according to lymphopenia or monocytopenia. Granuloma formation with BCG-coated beads (left) and CB-coated beads (right) was measured during 9 days in PBMCs from patients with severe sepsis. Patients were classified according to lymphopenia (A) and monocytopenia (B). The results are expressed as the percentage of beads entirely covered by PBMCs. The boxplots represent the medians with the first and third quartiles. The whiskers represent the highest value that is within 1.5*IQR. Data beyond the end of the whiskers are outliers and plotted as black points. * p < 0.05 represents the differences between patients with and without monocytopenia.

We previously reported that monocytes, but not lymphocytes, are required for granuloma formation [12]. Defective granuloma formation may be the result of the impaired recruitment of monocytes. We wondered if changes in monocyte and lymphocyte circulating levels impacted granuloma formation in patients with severe sepsis. The cohort of septic patients consisted of 11 patients with an abnormal formula, including 9 patients who did not form granulomas. The alteration of circulating mononuclear cells consisted of lymphopenia for 9 patients and monocytopenia for 5 patients. Three patients were both lymphopenic and monocytopenic. Lymphopenia in patients with severe sepsis was not significantly associated with the impaired formation of immune and innate granulomas although 7 patients with lymphopenia did not form granulomas (Fig 2A). In contrast, the five patients with monocytopenia (0.12 ± 0.08 × 10⁹ cells/l) were unable to form immune and innate granulomas (Fig 2B), showing that monocytopenia was associated with defective granuloma formation.

**Fig 3:**
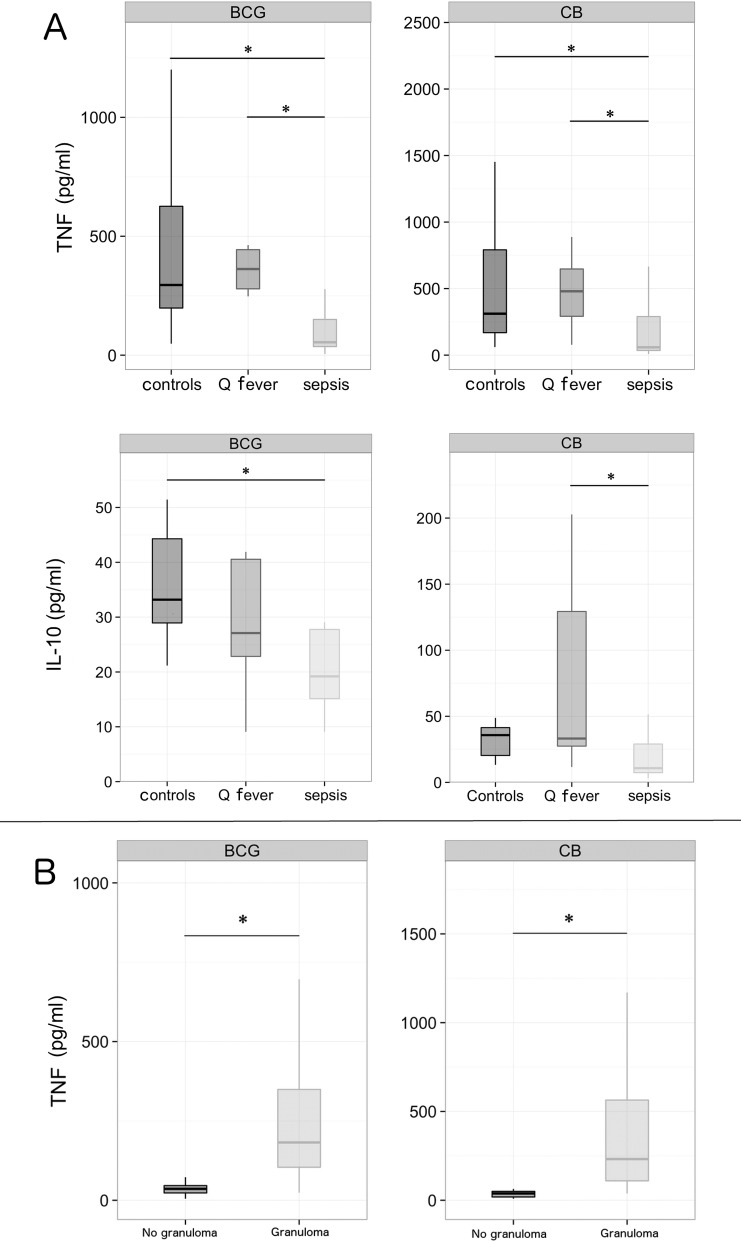
Cytokine production by PBMCs. PBMCs (2.5 × 10^5^ cells/well) isolated from patients with severe sepsis, cured Q fever and healthy controls were cultured in the presence of beads (50 beads/well) coated with BCG (left) or CB (right) extracts for 24 h. Each experiment was performed in duplicate. The presence of TNF and IL-10 in cell supernatants was assayed twice. The results were expressed as pg/ml. In A, TNF and IL-10 were measured in 8 Controls, 7 Q fever, and 14 samples of Sepsis. In B, TNF was measured in patients with severe sepsis who formed (6 samples) or did not form (8 samples) granulomas. The boxplots represent the medians with the first and third quartiles. The whiskers represent the highest value that is within 1.5 *IQR. * p < 0.05.

As monocytopenia did not account for all the patients with defective granuloma formation, we wondered whether alterations in cytokine imbalance interfered with granuloma formation. As TNF is associated with granuloma formation, we measured its release by PBMCs from septic patients. The amounts of TNF in supernatants from BCG-stimulated PBMCs were significantly (p = 0.02 and p = 0.03, respectively) lower in the patients with severe sepsis (54 [36–150] pg/ml) than in the healthy controls (295 [198–626] pg/ml) and the patients with cured Q fever (362 [279–443] pg/ml). Similarly, the amounts of TNF in supernatants from CB-stimulated PBMCs were significantly (p = 0.04 and p = 0.05, respectively) lower in the patients with severe sepsis (59 [35–290] pg/ml) than in the healthy controls (311 [169–791] pg/ml) and the patients with cured Q fever (480 [292–647] pg/ml) (Fig 3A). The decreased production of TNF may be related to the hyporesponsiveness of PBMCs from patients with severe sepsis or the increased production of an immunosuppressive cytokine such as IL-10. To discriminate between these hypotheses, we determined the release of IL-10 by PBMCs in the presence of BCG- or CB-coated beads. The release of IL-10 by control PBMCs stimulated with BCG- or CB-coated beads did not exceed 40 pg/ml in healthy controls and patients cured from Q fever. The release of IL-10 by PBMCs from septic patients was rather lower than that found in controls and patients with cured Q fever (Fig 3A), suggesting that the decreased release of TNF in severe sepsis was not related to the overproduction of an immunosuppressive cytokine such as IL-10. Finally, we studied the relationship between the capacity to form granulomas and the production of TNF in septic patients. The release of TNF (182 [104–349] and 232 [109–564] pg/ml, respectively) by PBMCs from the patients who formed immune (BCG) and innate (CB) granulomas was significantly (p < 0.05) higher than that of PBMCs from patients unable to form immune and innate granulomas (36 [23–46] and 38 [18–50] pg/ml, respectively) (Fig 3B). Taken together, these results showed that the defective formation of innate and immune granulomas in patients with severe sepsis was related to monocytopenia and associated with reduced production of TNF.

To tempt to relate defective granuloma formation and reduced production of TNF, we performed additional experiments. First, we added recombinant TNF to PBMCs from 3 patients who did not form granulomas: TNF was unable to increase the formation of immune and innate granulomas. Indeed, the percentage of immune granulomas observed when PBMCs were incubated in the presence of TNF was of 18 ± 25%; 27 ± 38%; 28 ± 42% at days 3, 6 and 9, respectively. Similarly, TNF did not increase the percentage of innate granulomas in patients unable to form granulomas (20 ± 25%, 27 ± 38%; 28 ± 42% at days 3, 6 and 9, respectively). Second, we added mAb directed against TNF to control PBMCs cultured for 9 days in the presence of beads coated with BCG or CB extracts. We found that such treatment had no effect on granuloma formation. Indeed, the percentage of immune granulomas remained high in the presence of anti-TNF mAb (55 ± 37%, 75 ± 35% and 87 ± 24% at days 3, 6 and 9, respectively. Similarly, anti-TNF mAb did not affect the percentage of innate granulomas (48 ± 42%, 78 ± 32% and 88 ± 21% at days 3, 6 and 9, respectively). Taken together, these results showed that the formation of granulomas was not directly related to TNF.

## Discussion

The study of immune response through the measurement of individual biomarkers to predict the evolution of patients with severe sepsis has been unsuccessful to date. We are proposing an alternative method, measurement of the *in vitro* formation of granulomas. It has been shown that PBMCs are able to form *in vitro* granulomas using beads covered with BCG [7] or CB [12] extracts. Here, we developed an *in vitro* assay to measure granuloma formation with low-size blood samples. In addition, using beads coated with BCG or CB extracts enabled us to investigate innate and immune responses in patients. The assay was distinct from the measurement of isolated biomarkers such as membrane antigens or soluble cytokines but could be related to whole blood stimulation assay. Nevertheless, while the former assesses activation and recruitment of PBMCs, the latter measures the ability of circulating cells to produce soluble mediators.

We found that granuloma formation was impaired in patients with severe sepsis. As the BCG vaccinal status of healthy subjects and septic patients was unknown, this may be a limit of the study. However, it is likely that most of them were immunized due to the inclusion criteria in France (affiliation to the national health system for being included in clinical trials). We also used samples of patients cured from Q fever as controls in order to have a population with specific immunization. The defective formation of granulomas with BCG-coated beads reflects a defective cell-mediated immune response. It may be related to loss of the delayed hypersensitivity response to common recall antigens associated with sepsis-induced immunosuppression [14]. Impaired granuloma formation with CB-coated beads in patients with severe sepsis who are not sensitized to CB suggests an immunoparalysis state [14]. Nevertheless, a minority of patients with severe sepsis was able to form granulomas, although we ignore if these granulomas are functionally competent. Conversely, one healthy control was unable to form granulomas. As the age of this control was 70 years, we suggest that the inability to form granulomas may be related to immunosenescence because about 10% of healthy aged individuals were unable to form granulomas (manuscript in preparation). Defective granuloma formation may be associated with the severity of sepsis or patient outcomes. We assessed granuloma formation according to severity including the severity score (SOFA) and survival. There is a tendency between defective granuloma formation and high SOFA but no significant correlation was found, likely due to the size of samples.

We then investigated the mechanisms involved in the defective granuloma formation in patients with severe sepsis. First, we showed that defective granuloma formation was associated with monocytopenia, but not with lymphopenia. This is consistent with our previous report in which we showed that monocytes, but not lymphocytes, are required for the initial phases of granuloma formation including the migration toward beads coated with bacterial extracts [12]. This may be also related to the role of macrophage supply in the maintain of granuloma integrity as demonstrated in a zebrafish model of granuloma formation [23]. Nevertheless, defective granuloma formation related to monocytopenia did not account for all the septic patients who did not form granulomas. Second, the defective granuloma formation in septic patients may depend on an inflammatory process or bacterial infection. We investigated the hypothesis based on cytokine imbalance because sepsis-induced immunosuppression is associated with decreased production of inflammatory cytokines and a high IL-10-to-TNF ratio [24]. We found that the TNF release induced by BCG or CB extracts was decreased in PBMCs from patients with severe sepsis. This finding is consistent with initial papers that showed the role of TNF in granuloma formation [5]. The decrease of TNF production occurs without IL-10 increase, suggesting that the modulation of TNF release was not related to the overproduction of IL-10, as in sepsis-induced immunosuppression based on LPS tolerance [25]. Our results are also distinct from those concerning patients with traumatic brain injury associated with defective granuloma formation in which a strong inflammatory response was observed [11]. The decrease in TNF release may result from a decreased number of TNF-producing cells in the blood, especially monocytes, rather than a repolarization of the immune response toward an anti-inflammatory profile. However, TNF adding to PBMCs from septic patients unable to form granulomas did not restore the formation of immune and innate granulomas. Similarly, adding anti-TNF mAb to control PBMCs did not inhibit the formation of immune and innate granulomas. These results suggest that TNF is dispensable or acts in synergy with other cytokines in granuloma formation. We hypothesize that the defective granuloma formation in septic patients may depend on bacterial infection. Indeed, Deknuydt *et al*. included patients with traumatic brain injury before the occurrence of nosocomial pneumonia and showed that impaired granuloma formation in these patients is associated with an increased frequency of secondary infections [11], suggesting that bacterial infection plays a major role in the defective formation of granulomas in patients with severe sepsis.

## Conclusion

In conclusion, this study showed that the assay of *in vitro* granuloma formation was convenient to study small blood samples in clinical practice. It also demonstrated that granuloma formation was impaired in a large proportion of patients with severe sepsis, as well as the role of monocytes. This study also suggests that immune response impairment is somewhat heterogeneous in patients with severe sepsis and use of this assay with larger cohorts of patients will be necessary.

## Supporting Information

S1 Fig Granuloma formation according to ICU discharge and SOFA score. PBMCs isolated from patients with severe sepsis were cultured in the presence of beads coated with BCG (left) or CB (right) extracts for 9 days. Patients were classified according to ICU discharge (dead or alive) (A) and SOFA score (≥ 7 or <7) (B). The results are expressed as the percentage of beads entirely covered by PBMCs. The boxplots represent the medians with the first and third quartiles. The whiskers represent the highest value that is within 1.5* IQR. Data beyond the end of the whiskers are outliers and plotted as black points.
(TIFF)

S1 Table Granuloma data. Represents each value (in percentage) of granuloma formation, at day 3, 6, 9, from controls, cured Q fever, or sepsis PBMCs, coated with CB or BCG beads, in duplicates.
(PDF)

S2 Table Cytokine data. Represents TNF and IL-10 values (in pg/ml) in cells supernatants for 1 day of culture, according granuloma formation, from controls, cured Q fever, or sepsis PBMCs, coated with CB or BCG beads, in duplicates.
(PDF)

S3 Table Patient features according to granuloma formation or no granuloma formation. SOFA: sequential organ failure assessment; SAPS: simplified acute physiology score; ICU: intensive care unit. Results are expressed as absolute number and percentage or median and interquartiles, as required.
(DOCX)

## References

[1] Zumla A, James DG. Granulomatous infections: etiology and classification. Clin Infect Dis Off Publ Infect Dis Soc Am. 1996; 23(1):146–58. 10.1093/clinids/23.1.146 8816144

[2] Molina-Ruiz AM, Requena L. Foreign Body Granulomas. Dermatol Clin. 2015; 33(3):497–523. 10.1016/j.det.2015.03.014 26143429

[3] Egen JG, Rothfuchs AG, Feng CG, Winter N, Sher A, Germain RN. Macrophage and T cell dynamics during the development and disintegration of mycobacterial granulomas. Immunity. 2008; 28(2):271–84. 10.1016/j.immuni.2007.12.010 18261937 PMC2390753

[4] Russell DG, Cardona PJ, Kim MJ, Allain S, Altare F. Foamy macrophages and the progression of the human TB granuloma. Nat Immunol. 2009; 10(9):943–8. 10.1038/ni.1781 19692995 PMC2759071

[5] Ramakrishnan L. Revisiting the role of the granuloma in tuberculosis. Nat Rev Immunol. 2012; 12(5):352–66. 10.1038/nri3211 22517424

[6] Shaler CR, Horvath CN, Jeyanathan M, Xing Z. Within the Enemy’s Camp: contribution of the granuloma to the dissemination, persistence and transmission of Mycobacterium tuberculosis. Front Immunol. 2013; 4:30 10.3389/fimmu.2013.00030 23420646 PMC3572501

[7] Puissegur MP, Botanch C, Duteyrat JL, Delsol G, Caratero C, Altare F. An in vitro dual model of mycobacterial granulomas to investigate the molecular interactions between mycobacteria and human host cells. Cell Microbiol. 2004; 6(5):423–33. 15056213 10.1111/j.1462-5822.2004.00371.x

[8] Delaby A, Espinosa L, Lépolard C, Capo C, Mège JL. 3D reconstruction of granulomas from transmitted light images implemented for long-time microscope applications. J Immunol Methods. 2010; 360(1–2):10–9. J Immunol Methods. 2010 Aug 31;360(1–2):10–9. 10.1016/j.jim.2010.06.008 20561526

[9] Meconi S, Vercellone A, Levillain F, Payré B, Al Saati T, Capilla F, et al Adherent-invasive Escherichia coli isolated from Crohn’s disease patients induce granulomas in vitro. Cell Microbiol. 2007; 9(5):1252–61. 17223928 10.1111/j.1462-5822.2006.00868.x

[10] Silva-Miranda M, Ekaza E, Breiman A, Asehnoune K, Barros-Aguirre D, Pethe K, et al High-content screening technology combined with a human granuloma model as a new approach to evaluate the activities of drugs against Mycobacterium tuberculosis. Antimicrob Agents Chemother. 2015; 59(1):693–7. 10.1128/AAC.03705-14 25348525 PMC4291390

[11] Deknuydt F, Roquilly A, Cinotti R, Altare F, Asehnoune K. An In vitro model of mycobacterial granuloma to investigate the immune response in brain-injured patients. Crit Care Med. 2013; 41(1):245–54. 10.1097/CCM.0b013e3182676052 23128384

[12] Delaby A, Gorvel L, Espinosa L, Lépolard C, Raoult D, Ghigo E, et al Defective monocyte dynamics in Q fever granuloma deficiency. J Infect Dis. 2012; 205(7):1086–94. 10.1093/infdis/jis013 22351939

[13] Calandra T, Cohen J, International Sepsis Forum Definition of Infection in the ICU Consensus Conference. The international sepsis forum consensus conference on definitions of infection in the intensive care unit. Crit Care Med. 2005; 33(7):1538–48. 16003060 10.1097/01.ccm.0000168253.91200.83

[14] Hotchkiss RS, Monneret G, Payen D. Sepsis-induced immunosuppression: from cellular dysfunctions to immunotherapy. Nat Rev Immunol. 2013; 13(12):862–74. 10.1038/nri3552 24232462 PMC4077177

[15] Debets JM, Kampmeijer R, van der Linden MP, Buurman WA, van der Linden CJ. Plasma tumor necrosis factor and mortality in critically ill septic patients. Crit Care Med. 1989; 17(6):489–94. 2721208 10.1097/00003246-198906000-00001

[16] Hotchkiss RS, Karl IE. The Pathophysiology and Treatment of Sepsis. N Engl J Med. 2003; 348(2):138–50. 12519925 10.1056/NEJMra021333

[17] Meisel C, Schefold JC, Pschowski R, Baumann T, Hetzger K, Gregor J, et al Granulocyte-macrophage colony-stimulating factor to reverse sepsis-associated immunosuppression: a double-blind, randomized, placebo-controlled multicenter trial. Am J Respir Crit Care Med. 2009; 180(7):640–8. 10.1164/rccm.200903-0363OC 19590022

[18] Leone M, Bechis C, Baumstarck K, Lefrant JY, Albanèse J, Jaber S, et al De-escalation versus continuation of empirical antimicrobial treatment in severe sepsis: a multicenter non-blinded randomized noninferiority trial. Intensive Care Med. 2014; 40(10):1399–408. 10.1007/s00134-014-3411-8 25091790

[19] Le Gall JR, Loirat P, Alperovitch A, Glaser P, Granthil C, Mathieu D, et al A simplified acute physiology score for ICU patients. Crit Care Med. 1984; 12(11):975–7. 6499483 10.1097/00003246-198411000-00012

[20] Vincent JL, Sakr Y, Sprung CL, Ranieri VM, Reinhart K, Gerlach H, et al Sepsis in European intensive care units: results of the SOAP study. Crit Care Med. 2006; 34(2):344–53. 16424713 10.1097/01.ccm.0000194725.48928.3a

[21] Honstettre A, Imbert G, Ghigo E, Gouriet F, Capo C, Raoult D, et al Dysregulation of cytokines in acute Q fever: role of interleukin-10 and tumor necrosis factor in chronic evolution of Q fever. J Infect Dis. 2003; 187(6):956–62. 12660942 10.1086/368129

[22] Gorvel L, Textoris J, Banchereau R, Ben Amara A, Tantibhedhyangkul W, von Bargen K, et al Intracellular bacteria interfere with dendritic cell functions: role of the type I interferon pathway. PloS One. 2014; 9:e99420 10.1371/journal.pone.0099420 24915541 PMC4051653

[23] Pagán AJ, Yang CT, Cameron J, Swaim LE, Ellett F, Lieschke GJ, et al Myeloid Growth Factors Promote Resistance to Mycobacterial Infection by Curtailing Granuloma Necrosis through Macrophage Replenishment. Cell Host Microbe. 2015; 18(1):15–26. 10.1016/j.chom.2015.06.008 26159717 PMC4509513

[24] Boomer JS, To K, Chang KC, Takasu O, Osborne DF, Walton AH, et al Immunosuppression in patients who die of sepsis and multiple organ failure. JAMA. 2011; 306(23):2594–605. 10.1001/jama.2011.1829 22187279 PMC3361243

[25] Biswas SK, Lopez-Collazo E. Endotoxin tolerance: new mechanisms, molecules and clinical significance. Trends Immunol. 2009; 30(10):475–87. 10.1016/j.it.2009.07.009 19781994

