# Supplementary figures and images for: Impaired Granuloma Formation in Sepsis: Impact of Monocytopenia
Source: PLoS One. 2016 Jul 21;11(7):e0158528. doi: 10.1371/journal.pone.0158528 (PMC4956217; doi:10.1371/journal.pone.0158528)

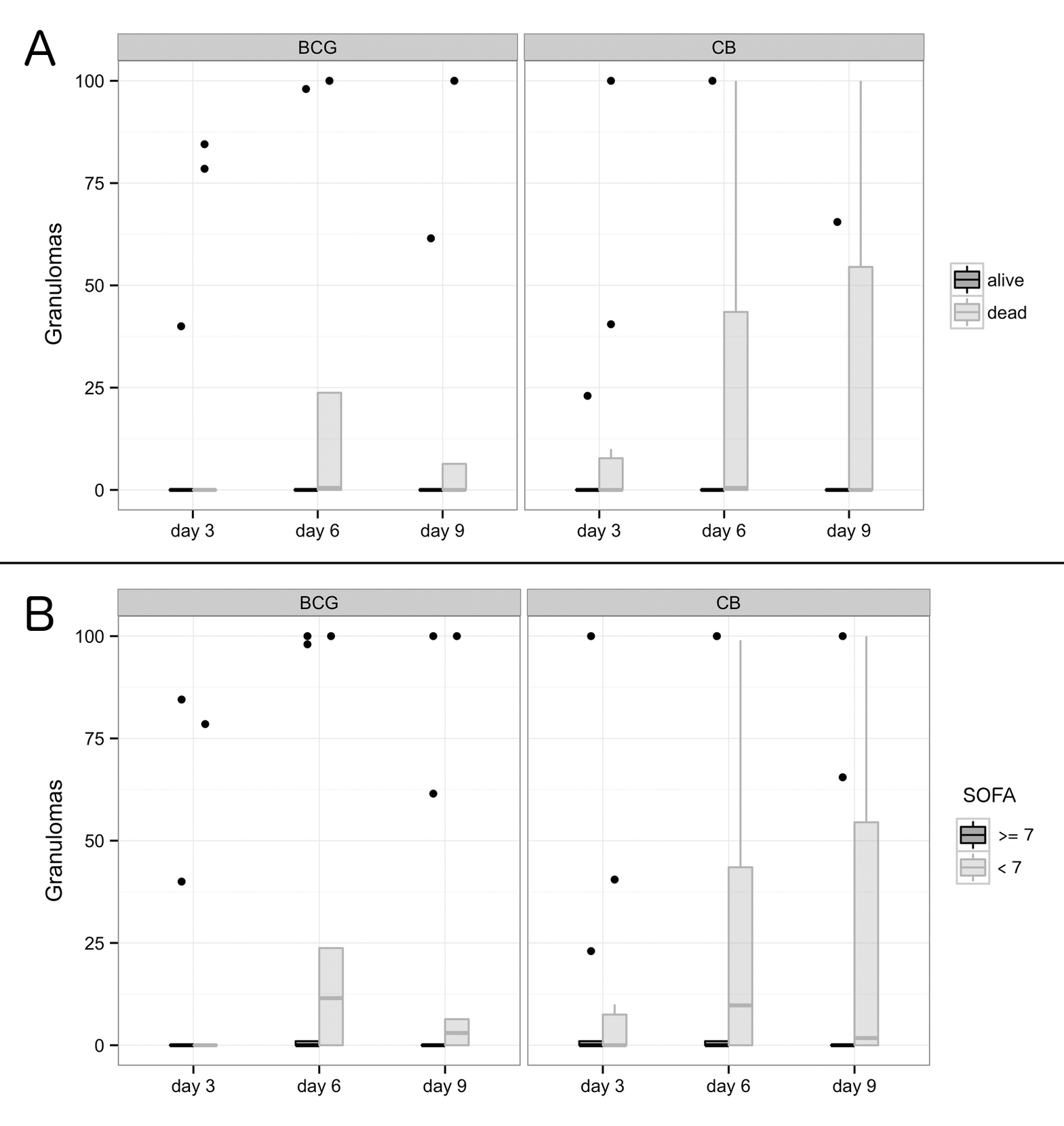

Supplement: S1 Fig — PBMCs isolated from patients with severe sepsis were cultured in the presence of beads coated with BCG (left) or CB (right) extracts for 9 days. Patients were classified according to ICU discharge (dead or alive) (A) and SOFA score (≥ 7 or <7) (B). The results are expressed as the percentage of beads entirely covered by PBMCs. The boxplots represent the medians with the first and third quartiles. The whiskers represent the highest value that is within 1.5* IQR. Data beyond the end of the whiskers are outliers and plotted as black points. (TIFF) [file pone.0158528.s001.tiff]
