# Supplementary material for: Impaired Granuloma Formation in Sepsis: Impact of Monocytopenia
Source: PLoS One. 2016 Jul 21;11(7):e0158528. doi: 10.1371/journal.pone.0158528 (PMC4956217; doi:10.1371/journal.pone.0158528)
Supplement: S1 Table — Represents each value (in percentage) of granuloma formation, at day 3, 6, 9, from controls, cured Q fever, or sepsis PBMCs, coated with CB or BCG beads, in duplicates. (PDF) [file pone.0158528.s002.pdf]

S1 Table. Granuloma Data

Group	Subject	Type	Day	Replicate	Value
controls	1	CB	day 3	1	7
controls	1	CB	day 3	2	100
controls	1	CB	day 6	1	54
controls	1	CB	day 6	2	88
controls	1	CB	day 9	1	NA
controls	1	CB	day 9	2	98
controls	1	BCG	day 3	1	97
controls	1	BCG	day 3	2	85
controls	1	BCG	day 6	1	NA
controls	1	BCG	day 6	2	90
controls	1	BCG	day 9	1	NA
controls	1	BCG	day 9	2	75
controls	2	CB	day 3	1	92
controls	2	CB	day 3	2	92
controls	2	CB	day 6	1	94
controls	2	CB	day 6	2	98
controls	2	CB	day 9	1	96
controls	2	CB	day 9	2	96
controls	2	BCG	day 3	1	40
controls	2	BCG	day 3	2	63
controls	2	BCG	day 6	1	82
controls	2	BCG	day 6	2	89
controls	2	BCG	day 9	1	66
controls	2	BCG	day 9	2	77
controls	3	CB	day 3	1	94
controls	3	CB	day 3	2	85
controls	3	CB	day 6	1	100
controls	3	CB	day 6	2	100
controls	3	CB	day 9	1	99
controls	3	CB	day 9	2	100
controls	3	BCG	day 3	1	48
controls	3	BCG	day 3	2	25
controls	3	BCG	day 6	1	89
controls	3	BCG	day 6	2	64
controls	3	BCG	day 9	1	94
controls	3	BCG	day 9	2	93
controls	4	CB	day 3	1	100
controls	4	CB	day 3	2	100
controls	4	CB	day 6	1	100
controls	4	CB	day 6	2	100
controls	4	CB	day 9	1	90
controls	4	CB	day 9	2	85
controls	4	BCG	day 3	1	100
controls	4	BCG	day 3	2	100
controls	4	BCG	day 6	1	100
controls	4	BCG	day 6	2	100
controls	4	BCG	day 9	1	97

controls	4	BCG	day 9	2	98
controls	5	CB	day 3	1	66
controls	5	CB	day 3	2	56
controls	5	CB	day 6	1	96
controls	5	CB	day 6	2	96
controls	5	CB	day 9	1	100
controls	5	CB	day 9	2	98
controls	5	BCG	day 3	1	63
controls	5	BCG	day 3	2	69
controls	5	BCG	day 6	1	94
controls	5	BCG	day 6	2	96
controls	5	BCG	day 9	1	98
controls	5	BCG	day 9	2	98
controls	6	CB	day 3	1	60
controls	6	CB	day 3	2	62
controls	6	CB	day 6	1	96
controls	6	CB	day 6	2	96
controls	6	CB	day 9	1	100
controls	6	CB	day 9	2	100
controls	6	BCG	day 3	1	64
controls	6	BCG	day 3	2	64
controls	6	BCG	day 6	1	99
controls	6	BCG	day 6	2	99
controls	6	BCG	day 9	1	100
controls	6	BCG	day 9	2	100
controls	7	CB	day 3	1	30
controls	7	CB	day 3	2	46
controls	7	CB	day 6	1	100
controls	7	CB	day 6	2	88
controls	7	CB	day 9	1	64
controls	7	CB	day 9	2	65
controls	7	BCG	day 3	1	45
controls	7	BCG	day 3	2	80
controls	7	BCG	day 6	1	88
controls	7	BCG	day 6	2	100
controls	7	BCG	day 9	1	89
controls	7	BCG	day 9	2	96
controls	8	CB	day 3	1	70
controls	8	CB	day 3	2	89
controls	8	CB	day 6	1	100
controls	8	CB	day 6	2	100
controls	8	CB	day 9	1	100
controls	8	CB	day 9	2	100
controls	8	BCG	day 3	1	95
controls	8	BCG	day 3	2	92
controls	8	BCG	day 6	1	100
controls	8	BCG	day 6	2	100
controls	8	BCG	day 9	1	100
controls	8	BCG	day 9	2	100

controls	9	CB	day 3	1	68
controls	9	CB	day 3	2	74
controls	9	CB	day 6	1	100
controls	9	CB	day 6	2	100
controls	9	CB	day 9	1	100
controls	9	CB	day 9	2	100
controls	9	BCG	day 3	1	86
controls	9	BCG	day 3	2	87
controls	9	BCG	day 6	1	99
controls	9	BCG	day 6	2	99
controls	9	BCG	day 9	1	100
controls	9	BCG	day 9	2	100
controls	10	CB	day 3	1	51
controls	10	CB	day 3	2	27
controls	10	CB	day 6	1	77
controls	10	CB	day 6	2	58
controls	10	CB	day 9	1	80
controls	10	CB	day 9	2	45
controls	10	BCG	day 3	1	21
controls	10	BCG	day 3	2	37
controls	10	BCG	day 6	1	25
controls	10	BCG	day 6	2	51
controls	10	BCG	day 9	1	22
controls	10	BCG	day 9	2	56
controls	11	CB	day 3	1	8
controls	11	CB	day 3	2	NA
controls	11	CB	day 6	1	20
controls	11	CB	day 6	2	NA
controls	11	CB	day 9	1	6
controls	11	CB	day 9	2	NA
controls	11	BCG	day 3	1	4
controls	11	BCG	day 3	2	NA
controls	11	BCG	day 6	1	3
controls	11	BCG	day 6	2	NA
controls	11	BCG	day 9	1	1
controls	11	BCG	day 9	2	NA
controls	12	CB	day 3	1	7
controls	12	CB	day 3	2	7
controls	12	CB	day 6	1	54
controls	12	CB	day 6	2	88
controls	12	CB	day 9	1	NA
controls	12	CB	day 9	2	98
controls	12	BCG	day 3	1	97
controls	12	BCG	day 3	2	85
controls	12	BCG	day 6	1	NA
controls	12	BCG	day 6	2	90
controls	12	BCG	day 9	1	NA
controls	12	BCG	day 9	2	75
cured Q fever	1	CB	day 3	1	100

cured Q fever	1	CB	day 3	2	100
cured Q fever	1	CB	day 6	1	100
cured Q fever	1	CB	day 6	2	100
cured Q fever	1	CB	day 9	1	95
cured Q fever	1	CB	day 9	2	95
cured Q fever	1	BCG	day 3	1	100
cured Q fever	1	BCG	day 3	2	100
cured Q fever	1	BCG	day 6	1	100
cured Q fever	1	BCG	day 6	2	100
cured Q fever	1	BCG	day 9	1	95
cured Q fever	1	BCG	day 9	2	100
cured Q fever	2	CB	day 3	1	100
cured Q fever	2	CB	day 3	2	100
cured Q fever	2	CB	day 6	1	100
cured Q fever	2	CB	day 6	2	100
cured Q fever	2	CB	day 9	1	98
cured Q fever	2	CB	day 9	2	98
cured Q fever	2	BCG	day 3	1	100
cured Q fever	2	BCG	day 3	2	100
cured Q fever	2	BCG	day 6	1	100
cured Q fever	2	BCG	day 6	2	100
cured Q fever	2	BCG	day 9	1	99
cured Q fever	2	BCG	day 9	2	100
cured Q fever	3	CB	day 3	1	27
cured Q fever	3	CB	day 3	2	2
cured Q fever	3	CB	day 6	1	100
cured Q fever	3	CB	day 6	2	100
cured Q fever	3	CB	day 9	1	98
cured Q fever	3	CB	day 9	2	100
cured Q fever	3	BCG	day 3	1	3
cured Q fever	3	BCG	day 3	2	9
cured Q fever	3	BCG	day 6	1	96
cured Q fever	3	BCG	day 6	2	95
cured Q fever	3	BCG	day 9	1	100
cured Q fever	3	BCG	day 9	2	100
cured Q fever	4	CB	day 3	1	0
cured Q fever	4	CB	day 3	2	0
cured Q fever	4	CB	day 3	3	0
cured Q fever	4	CB	day 6	1	96
cured Q fever	4	CB	day 6	2	98
cured Q fever	4	CB	day 6	3	98
cured Q fever	4	CB	day 9	1	95
cured Q fever	4	CB	day 9	2	98
cured Q fever	4	CB	day 9	3	96
cured Q fever	4	BCG	day 3	1	3
cured Q fever	4	BCG	day 3	2	0
cured Q fever	4	BCG	day 6	1	94
cured Q fever	4	BCG	day 6	2	98
cured Q fever	4	BCG	day 9	1	98

cured Q fever	4	BCG	day 9	2	98
cured Q fever	5	CB	day 3	1	100
cured Q fever	5	CB	day 3	2	100
cured Q fever	5	CB	day 6	1	100
cured Q fever	5	CB	day 6	2	100
cured Q fever	5	CB	day 9	1	100
cured Q fever	5	CB	day 9	2	100
cured Q fever	5	BCG	day 3	1	100
cured Q fever	5	BCG	day 3	2	100
cured Q fever	5	BCG	day 6	1	100
cured Q fever	5	BCG	day 6	2	100
cured Q fever	5	BCG	day 9	1	100
cured Q fever	5	BCG	day 9	2	100
cured Q fever	6	CB	day 3	1	100
cured Q fever	6	CB	day 3	2	100
cured Q fever	6	CB	day 6	1	100
cured Q fever	6	CB	day 6	2	100
cured Q fever	6	CB	day 9	1	100
cured Q fever	6	CB	day 9	2	100
cured Q fever	6	BCG	day 3	1	100
cured Q fever	6	BCG	day 3	2	100
cured Q fever	6	BCG	day 6	1	100
cured Q fever	6	BCG	day 6	2	100
cured Q fever	6	BCG	day 9	1	100
cured Q fever	6	BCG	day 9	2	100
cured Q fever	7	CB	day 3	1	98
cured Q fever	7	CB	day 3	2	98
cured Q fever	7	CB	day 6	1	100
cured Q fever	7	CB	day 6	2	100
cured Q fever	7	CB	day 9	1	100
cured Q fever	7	CB	day 9	2	100
cured Q fever	7	BCG	day 3	1	98
cured Q fever	7	BCG	day 3	2	98
cured Q fever	7	BCG	day 6	1	100
cured Q fever	7	BCG	day 6	2	100
cured Q fever	7	BCG	day 9	1	100
cured Q fever	7	BCG	day 9	2	100
cured Q fever	8	CB	day 3	1	100
cured Q fever	8	CB	day 3	2	100
cured Q fever	8	CB	day 6	1	100
cured Q fever	8	CB	day 6	2	100
cured Q fever	8	CB	day 9	1	100
cured Q fever	8	CB	day 9	2	100
cured Q fever	8	BCG	day 3	1	100
cured Q fever	8	BCG	day 3	2	80
cured Q fever	8	BCG	day 6	1	100
cured Q fever	8	BCG	day 6	2	98
cured Q fever	8	BCG	day 9	1	100
cured Q fever	8	BCG	day 9	2	100

cured Q fever	9	CB	day 3	1		100	
cured Q fever	9	CB	day 3	2		100	
cured Q fever	9	CB	day 6	1		100	
cured Q fever	9	CB	day 6	2		100	
cured Q fever	9	CB	day 9	1		100	
cured Q fever	9	CB	day 9	2		100	
cured Q fever	9	BCG	day 3	1		74	
cured Q fever	9	BCG	day 3	2		76	
cured Q fever	9	BCG	day 6	1		100	
cured Q fever	9	BCG	day 6	2		99	
cured Q fever	9	BCG	day 9	1		100	
cured Q fever	9	BCG	day 9	2		100	
sepsis	1	CB	day 3	1		0	yes
sepsis	1	CB	day 3	2	NA		yes
sepsis	1	CB	day 6	1		0	yes
sepsis	1	CB	day 6	2	NA		yes
sepsis	1	CB	day 9	1		0	yes
sepsis	1	CB	day 9	2	NA		yes
sepsis	1	BCG	day 3	1		0	yes
sepsis	1	BCG	day 3	2	NA		yes
sepsis	1	BCG	day 6	1		0	yes
sepsis	1	BCG	day 6	2	NA		yes
sepsis	1	BCG	day 9	1		0	yes
sepsis	1	BCG	day 9	2	NA		yes
sepsis	2	CB	day 3	1		1	no
sepsis	2	CB	day 3	2	NA		no
sepsis	2	CB	day 6	1		1	no
sepsis	2	CB	day 6	2	NA		no
sepsis	2	CB	day 9	1		0	no
sepsis	2	CB	day 9	2	NA		no
sepsis	2	BCG	day 3	1		0	no
sepsis	2	BCG	day 3	2	NA		no
sepsis	2	BCG	day 6	1		1	no
sepsis	2	BCG	day 6	2	NA		no
sepsis	2	BCG	day 9	1		0	no
sepsis	2	BCG	day 9	2	NA		no
sepsis	3	CB	day 3	1		0	yes
sepsis	3	CB	day 3	2		0	yes
sepsis	3	CB	day 6	1		0	yes
sepsis	3	CB	day 6	2		0	yes
sepsis	3	CB	day 9	1		0	yes
sepsis	3	CB	day 9	2		0	yes
sepsis	3	BCG	day 3	1		0	yes
sepsis	3	BCG	day 3	2		0	yes
sepsis	3	BCG	day 6	1		1	yes
sepsis	3	BCG	day 6	2		0	yes
sepsis	3	BCG	day 9	1		0	yes
sepsis	3	BCG	day 9	2		0	yes
sepsis	4	CB	day 3	1		100	no

sepsis	4	CB	day 3	2	100	no
sepsis	4	CB	day 6	1	100	no
sepsis	4	CB	day 6	2	100	no
sepsis	4	CB	day 6	3	100	no
sepsis	4	CB	day 9	1	100	no
sepsis	4	CB	day 9	2	100	no
sepsis	4	BCG	day 3	1	87	no
sepsis	4	BCG	day 3	2	82	no
sepsis	4	BCG	day 6	1	100	no
sepsis	4	BCG	day 6	2	100	no
sepsis	4	BCG	day 9	1	100	no
sepsis	4	BCG	day 9	2	100	no
sepsis	5	CB	day 3	1	0	no
sepsis	5	CB	day 3	2	NA	no
sepsis	5	CB	day 6	1	0	no
sepsis	5	CB	day 6	2	NA	no
sepsis	5	CB	day 9	1	0	no
sepsis	5	CB	day 9	2	NA	no
sepsis	5	BCG	day 3	1	0	no
sepsis	5	BCG	day 3	2	NA	no
sepsis	5	BCG	day 6	1	0	no
sepsis	5	BCG	day 6	2	NA	no
sepsis	5	BCG	day 9	1	0	no
sepsis	5	BCG	day 9	2	NA	no
sepsis	6	CB	day 3	1	0	yes
sepsis	6	CB	day 3	2	NA	yes
sepsis	6	CB	day 6	1	0	yes
sepsis	6	CB	day 6	2	NA	yes
sepsis	6	CB	day 9	1	0	yes
sepsis	6	CB	day 9	2	NA	yes
sepsis	6	BCG	day 3	1	0	yes
sepsis	6	BCG	day 3	2	NA	yes
sepsis	6	BCG	day 6	1	0	yes
sepsis	6	BCG	day 6	2	NA	yes
sepsis	6	BCG	day 9	1	0	yes
sepsis	6	BCG	day 9	2	NA	yes
sepsis	7	CB	day 3	1	11	no
sepsis	7	CB	day 3	2	9	no
sepsis	7	CB	day 6	1	47	no
sepsis	7	CB	day 6	2	56	no
sepsis	7	CB	day 9	1	63	no
sepsis	7	CB	day 9	2	80	no
sepsis	7	BCG	day 3	1	0	no
sepsis	7	BCG	day 3	2	0	no
sepsis	7	BCG	day 6	1	30	no
sepsis	7	BCG	day 6	2	18	no
sepsis	7	BCG	day 9	1	11	no
sepsis	7	BCG	day 9	2	2	no
sepsis	8	CB	day 3	1	0	no

sepsis	8	CB	day 3	2	NA	no
sepsis	8	CB	day 6	1	0	no
sepsis	8	CB	day 6	2	NA	no
sepsis	8	CB	day 9	1	0	no
sepsis	8	CB	day 9	2	NA	no
sepsis	8	BCG	day 3	1	0	no
sepsis	8	BCG	day 3	2	NA	no
sepsis	8	BCG	day 6	1	0	no
sepsis	8	BCG	day 6	2	NA	no
sepsis	8	BCG	day 9	1	0	no
sepsis	8	BCG	day 9	2	NA	no
sepsis	9	CB	day 3	1	0	no
sepsis	9	CB	day 3	2	0	no
sepsis	9	CB	day 6	1	0	no
sepsis	9	CB	day 6	2	0	no
sepsis	9	CB	day 9	1	0	no
sepsis	9	CB	day 9	2	0	no
sepsis	9	BCG	day 3	1	0	no
sepsis	9	BCG	day 3	2	0	no
sepsis	9	BCG	day 6	1	0	no
sepsis	9	BCG	day 6	2	0	no
sepsis	9	BCG	day 9	1	0	no
sepsis	9	BCG	day 9	2	0	no
sepsis	10	CB	day 3	1	100	no
sepsis	10	CB	day 3	2	100	no
sepsis	10	CB	day 6	1	100	no
sepsis	10	CB	day 6	2	100	no
sepsis	10	CB	day 9	1	100	no
sepsis	10	CB	day 9	2	100	no
sepsis	10	BCG	day 3	1	100	no
sepsis	10	BCG	day 3	2	100	no
sepsis	10	BCG	day 6	1	100	no
sepsis	10	BCG	day 6	2	100	no
sepsis	10	BCG	day 9	1	100	no
sepsis	10	BCG	day 9	2	100	no
sepsis	11	CB	day 3	1	0	yes
sepsis	11	CB	day 3	2	0	yes
sepsis	11	CB	day 6	1	0	yes
sepsis	11	CB	day 6	2	0	yes
sepsis	11	CB	day 9	1	0	yes
sepsis	11	CB	day 9	2	0	yes
sepsis	11	BCG	day 3	1	0	yes
sepsis	11	BCG	day 3	2	0	yes
sepsis	11	BCG	day 6	1	0	yes
sepsis	11	BCG	day 6	2	0	yes
sepsis	11	BCG	day 9	1	0	yes
sepsis	11	BCG	day 9	2	0	yes
sepsis	12	CB	day 3	1	0	yes
sepsis	12	CB	day 3	2	NA	yes

sepsis	12	CB	day 6	1	0	yes
sepsis	12	CB	day 6	2	NA	yes
sepsis	12	CB	day 9	1	0	yes
sepsis	12	CB	day 9	2	NA	yes
sepsis	12	BCG	day 3	1	0	yes
sepsis	12	BCG	day 3	2	NA	yes
sepsis	12	BCG	day 6	1	0	yes
sepsis	12	BCG	day 6	2	NA	yes
sepsis	12	BCG	day 9	1	0	yes
sepsis	12	BCG	day 9	2	NA	yes
sepsis	13	CB	day 3	1	0	no
sepsis	13	CB	day 3	2	NA	no
sepsis	13	CB	day 6	1	1	no
sepsis	13	CB	day 6	2	NA	no
sepsis	13	CB	day 9	1	13	no
sepsis	13	CB	day 9	2	NA	no
sepsis	13	BCG	day 3	1	5	no
sepsis	13	BCG	day 3	2	NA	no
sepsis	13	BCG	day 6	1	29	no
sepsis	13	BCG	day 6	2	NA	no
sepsis	13	BCG	day 9	1	48	no
sepsis	13	BCG	day 9	2	NA	no
sepsis	14	CB	day 3	1	0	no
sepsis	14	CB	day 3	2	0	no
sepsis	14	CB	day 6	1	0	no
sepsis	14	CB	day 6	2	0	no
sepsis	14	CB	day 9	1	0	no
sepsis	14	CB	day 9	2	0	no
sepsis	14	BCG	day 3	1	0	no
sepsis	14	BCG	day 3	2	0	no
sepsis	14	BCG	day 6	1	0	no
sepsis	14	BCG	day 6	2	0	no
sepsis	14	BCG	day 9	1	0	no
sepsis	14	BCG	day 9	2	0	no
sepsis	15	CB	day 3	1	46	no
sepsis	15	CB	day 3	2	35	no
sepsis	15	CB	day 6	1	99	no
sepsis	15	CB	day 6	2	99	no
sepsis	15	CB	day 9	1	100	no
sepsis	15	CB	day 9	2	100	no
sepsis	15	BCG	day 3	1	66	no
sepsis	15	BCG	day 3	2	91	no
sepsis	15	BCG	day 6	1	100	no
sepsis	15	BCG	day 6	2	100	no
sepsis	15	BCG	day 9	1	100	no
sepsis	15	BCG	day 9	2	100	no
sepsis	16	CB	day 3	1	0	no
sepsis	16	CB	day 3	2	0	no
sepsis	16	CB	day 6	1	15	no

sepsis	16	CB	day 6	2	24	no
sepsis	16	CB	day 9	1	1	no
sepsis	16	CB	day 9	2	6	no
sepsis	16	BCG	day 3	1	0	no
sepsis	16	BCG	day 3	2	0	no
sepsis	16	BCG	day 6	1	25	no
sepsis	16	BCG	day 6	2	21	no
sepsis	16	BCG	day 9	1	7	no
sepsis	16	BCG	day 9	2	5	no
sepsis	17	CB	day 3	1	14	yes
sepsis	17	CB	day 3	2	32	yes
sepsis	17	CB	day 6	1	100	yes
sepsis	17	CB	day 6	2	100	yes
sepsis	17	CB	day 9	1	57	yes
sepsis	17	CB	day 9	2	74	yes
sepsis	17	BCG	day 3	1	45	yes
sepsis	17	BCG	day 3	2	35	yes
sepsis	17	BCG	day 6	1	100	yes
sepsis	17	BCG	day 6	2	96	yes
sepsis	17	BCG	day 9	1	68	yes
sepsis	17	BCG	day 9	2	55	yes
sepsis	18	CB	day 3	1	0	no
sepsis	18	CB	day 3	2	0	no
sepsis	18	CB	day 6	1	0	no
sepsis	18	CB	day 6	2	0	no
sepsis	18	CB	day 9	1	0	no
sepsis	18	CB	day 9	2	0	no
sepsis	18	BCG	day 3	1	0	no
sepsis	18	BCG	day 3	2	0	no
sepsis	18	BCG	day 6	1	0	no
sepsis	18	BCG	day 6	2	0	no
sepsis	18	BCG	day 9	1	0	no
sepsis	18	BCG	day 9	2	0	no
sepsis	19	CB	day 3	1	0	no
sepsis	19	CB	day 3	2	NA	no
sepsis	19	CB	day 6	1	0	no
sepsis	19	CB	day 6	2	NA	no
sepsis	19	CB	day 9	1	0	no
sepsis	19	CB	day 9	2	0	no
sepsis	19	BCG	day 3	1	0	no
sepsis	19	BCG	day 3	2	NA	no
sepsis	19	BCG	day 6	1	0	no
sepsis	19	BCG	day 6	2	NA	no
sepsis	19	BCG	day 9	1	0	no
sepsis	19	BCG	day 9	2	NA	no

6	no	no
6	no	no
6	no	no
6	no	no
6	no	no
6	no	no
6	no	no
6	no	no
6	no	no
6	no	no
6	no	no
6	no	no
8	yes	no
8	yes	no
8	yes	no
8	yes	no
8	yes	no
8	yes	no
8	yes	no
8	yes	no
8	yes	no
8	yes	no
8	yes	no
8	yes	no
	yes	yes
	yes	yes
	yes	yes
	yes	yes
	yes	yes
	yes	yes
	yes	yes
	yes	yes
	yes	yes
	yes	yes
	yes	yes
	yes	yes
9	yes	no

9	yes	no
9	yes	no
9	yes	no
9	yes	no
9	yes	no
9	yes	no
9	yes	no
9	yes	no
9	yes	no
9	yes	no
9	yes	no
9	yes	no
7	no	no
7	no	no
7	no	no
7	no	no
7	no	no
7	no	no
7	no	no
7	no	no
7	no	no
7	no	no
7	no	no
7	no	no
12	yes	no
12	yes	no
12	yes	no
12	yes	no
12	yes	no
12	yes	no
12	yes	no
12	yes	no
12	yes	no
12	yes	no
12	yes	no
12	yes	no
3	no	no
3	no	no
3	no	no
3	no	no
3	no	no
3	no	no
3	no	no
3	no	no
3	no	no
3	no	no
3	no	no
3	no	no
15	no	no

15	no	no
15	no	no
15	no	no
15	no	no
15	no	no
15	no	no
15	no	no
15	no	no
15	no	no
15	no	no
15	no	no
	yes	yes
	yes	yes
	yes	yes
	yes	yes
	yes	yes
	yes	yes
	yes	yes
	yes	yes
	yes	yes
	yes	yes
	yes	yes
	yes	yes
	yes	no
	yes	no
	yes	no
	yes	no
	yes	no
	yes	no
	yes	no
	yes	no
	yes	no
	yes	no
	yes	no
	yes	no
10	yes	no
10	yes	no
10	yes	no
10	yes	no
10	yes	no
10	yes	no
10	yes	no
10	yes	no
10	yes	no
10	yes	no
10	yes	no
10	yes	no
10	no	yes
10	no	yes

10	no	yes
10	no	yes
10	no	yes
10	no	yes
10	no	yes
10	no	yes
10	no	yes
10	no	yes
10	no	yes
10	no	yes
	no	no
	no	no
	no	no
	no	no
	no	no
	no	no
	no	no
	no	no
	no	no
	no	no
	no	no
	no	no
5	no	no
5	no	no
5	no	no
5	no	no
5	no	no
5	no	no
5	no	no
5	no	no
5	no	no
5	no	no
5	no	no
5	no	no
6	no	no
6	no	no
6	no	no
6	no	no
6	no	no
6	no	no
6	no	no
6	no	no
6	no	no
6	no	no
6	no	no
6	no	no
6	yes	yes
6	yes	yes
6	yes	yes

6	yes	yes
6	yes	yes
6	yes	yes
6	yes	yes
6	yes	yes
6	yes	yes
6	yes	yes
6	yes	yes
6	yes	yes
10	no	no
10	no	no
10	no	no
10	no	no
10	no	no
10	no	no
10	no	no
10	no	no
10	no	no
10	no	no
10	no	no
10	no	no
12	yes	no
12	yes	no
12	yes	no
12	yes	no
12	yes	no
12	yes	no
12	yes	no
12	yes	no
12	yes	no
12	yes	no
12	yes	no
12	yes	no
2	no	yes
2	no	yes
2	no	yes
2	no	yes
2	no	yes
2	no	yes
2	no	yes
2	no	yes
2	no	yes
2	no	yes
2	no	yes
2	no	yes
