# Supplementary material for: Impaired Granuloma Formation in Sepsis: Impact of Monocytopenia
Source: PLoS One. 2016 Jul 21;11(7):e0158528. doi: 10.1371/journal.pone.0158528 (PMC4956217; doi:10.1371/journal.pone.0158528)
Supplement: S2 Table — Represents TNF and IL-10 values (in pg/ml) in cells supernatants for 1 day of culture, according granuloma formation, from controls, cured Q fever, or sepsis PBMCs, coated with CB or BCG beads, in duplicates. (PDF) [file pone.0158528.s003.pdf]

Group	Subjects	Granulomas	Type	IL10	TNF
sepsis	1	yes	CB	5,772	83,99375
sepsis	1	yes	BCG	NA	54,14
sepsis	2	no	CB	3,178	115,61325
sepsis	2	no	BCG	21,4632	4,8231
sepsis	3	no	CB	NA	35,285
sepsis	3	no	BCG	NA	NA
sepsis	4	yes	CB	51,58	665,74365
sepsis	4	yes	BCG	28,9924	149,9964
sepsis	5	yes	CB	91,0279	573,5594
sepsis	5	yes	BCG	84,3415	464,1182
sepsis	6	NA	CB	NA	NA
sepsis	6	NA	BCG	NA	NA
sepsis	7	no	CB	8,2837	46,2518
sepsis	7	no	BCG	9,1195	49,5682
sepsis	8	NA	CB	NA	NA
sepsis	8	NA	BCG	NA	NA
sepsis	9	NA	CB	NA	NA
sepsis	9	NA	BCG	NA	NA
sepsis	10	yes	CB	29,019	289,8275
sepsis	10	yes	BCG	26,52825	277,2575
sepsis	11	no	CB	23,148	8,445
sepsis	11	no	BCG	19,2009	40,49
sepsis	12	no	CB	7,3596	13,97
sepsis	12	no	BCG	15,2538	36,07
sepsis	13	yes	CB	10,7535	58,85375
sepsis	13	yes	BCG	NA	94,9925
sepsis	14	NA	CB	NA	NA
sepsis	14	NA	BCG	NA	NA
sepsis	15	yes	CB	37,5972	971,37165
sepsis	15	yes	BCG	15,0096	237,86445
sepsis	16	no	CB	7,4479	51,2264
sepsis	16	no	BCG	16,6417	72,783
sepsis	17	NA	CB	NA	NA
sepsis	17	NA	BCG	NA	NA
sepsis	18	no	CB	3,4125	15,075
sepsis	18	no	BCG	29,06865	36,07
sepsis	19	no	CB	16,0852	
sepsis	19	no	BCG	11,7828	4,8231
cured Q fever	1	NA	CB	NA	NA
cured Q fever	1	NA	BCG	NA	NA
cured Q fever	2	NA	CB	NA	NA
cured Q fever	2	NA	BCG	NA	NA
cured Q fever	3	yes	CB	413,9109	342,155
cured Q fever	3	yes	BCG	305,36565	247,125
cured Q fever	4	yes	CB	202,74105	78,06
cured Q fever	4	yes	BCG	27,0951	23,915
cured Q fever	5	yes	CB	27,3585	888,47375
cured Q fever	5	yes	BCG	9,093	362,105

cured Q fever	6	yes	CB	33,17025	479,94875
cured Q fever	6	yes	BCG	24,86775	462,665
cured Q fever	7	yes	CB	55,8824	784,1745
cured Q fever	7	yes	BCG	41,8996	310,4511
cured Q fever	8	yes	CB	11,6269	241,9194
cured Q fever	8	yes	BCG	20,8207	425,9796
cured Q fever	9	yes	CB	27,5071	510,5478
cured Q fever	9	yes	BCG	39,2083	460,8018
controls	1	yes	CB	48,80415	97,95
controls	1	yes	BCG	33,01575	50,435
controls	2	yes	CB	13,2985	296,64
controls	2	yes	BCG	33,3577	308,2474
controls	3	yes	CB	34,9893	60,38
controls	3	yes	BCG	21,17445	48,225
controls	4	yes	CB	20,8207	192,1734
controls	4	yes	BCG	30,8503	281,7162
controls	5	yes	CB	19,056	571,08125
controls	5	yes	BCG	23,20725	434,3825
controls	6	yes	CB	36,5216	2384,90115
controls	6	yes	BCG	41,8996	1334,3049
controls	7	yes	CB	38,982	325,96625
controls	7	yes	BCG	51,43575	247,40375
controls	8	NA	CB	NA	NA
controls	8	NA	BCG	NA	NA
controls	9	NA	CB	NA	NA
controls	9	NA	BCG	NA	NA
controls	10	yes	CB	89,226	1452,73575
controls	10	yes	BCG	125,7964	1200,59265
controls	11	no	CB	NA	NA
controls	11	no	BCG	NA	NA
controls	12	no	CB	NA	NA
controls	12	no	BCG	NA	NA
