# Supplementary material for: Impaired Granuloma Formation in Sepsis: Impact of Monocytopenia
Source: PLoS One. 2016 Jul 21;11(7):e0158528. doi: 10.1371/journal.pone.0158528 (PMC4956217; doi:10.1371/journal.pone.0158528)
Supplement: S3 Table — SOFA: sequential organ failure assessment; SAPS: simplified acute physiology score; ICU: intensive care unit. Results are expressed as absolute number and percentage or median and interquartiles, as required. (DOCX) [file pone.0158528.s004.docx]

**S3 Table. Patient features according according to granuloma formation or no granuloma formation.**

| **Clinical features** | **Granulomas** | **No granulomas** |
| --- | --- | --- |
| Patients (Males/Females) | 13 (11/2) | 6 (6/0) |
| Age [Years] | 57 [50-70] | 54 [46-61] |
| SAPS II | 40 [31-45] | 37 [32-49] |
| SOFA | 8 [6-11] | 8 [5-9] |
| Number of ICU days | 12 [6-16] | 13 [9-18] |
| Mechanical ventilation (days) | 7 [4-10] | 13 [9-26] |
| Vasopressors use (days) | 3 [3-5] | 4 [2-6] |
| ICU Mortality (%) | 5 (38) | 1 (17) |
| Normal formula (%) | 4 (31) | 4 (67) |
| Lymphopenic (%) | 7 (54) | 2 (33) |
| Monocytopenic (%) | 5 (38) | 0 (0) |

SOFA: sequential organ failure assessment; SAPS: simplified acute physiology score; ICU: intensive care unit. Results are expressed as absolute number and percentage or median and interquartiles, as required.
